# The Role of MET Inhibitor Therapies in the Treatment of Advanced Non-Small Cell Lung Cancer

**DOI:** 10.3390/jcm9061918

**Published:** 2020-06-19

**Authors:** Ramon Andrade De Mello, Nathália Moisés Neves, Giovanna Araújo Amaral, Estela Gudin Lippo, Pedro Castelo-Branco, Daniel Humberto Pozza, Carla Chizuru Tajima, Georgios Antoniou

**Affiliations:** 1 Algarve Biomedical Centre, Department of Biomedical Sciences and Medicine University of Algarve (DCBM UALG), 8005-139 Faro, Portugal; castelobranco.pedro@gmail.com; 2 Division of Medical Oncology, Escola Paulista de Medicina, Federal University of São Paulo (UNIFESP), São Paulo 04037-004, Brazil; nathalia.neves@unifesp.br (N.M.N.); gioaraujoamaral@gmail.com (G.A.A.); 3 Precision Oncology and Health Economics Group (ONCOPRECH), Post-Graduation Program in Medicine, Nine of July University (UNINOVE), São Paulo 01525-000, Brazil; 4 School of Biomedical Sciences, Santo Amaro University, São Paulo 01525-000, Brazil; estelaglippo@gmail.com; 5 Department of Biomedicine & I3S, Faculty of Medicine, University of Porto (FMUP), 4200-317 Porto, Portugal; dhpozza@gmail.com; 6 Hospital São José & Hospital São Joaquim, A Beneficência Portuguesa de São Paulo, São Paulo 01323-001, Brazil; carlatajima@yahoo.com.br; 7 Division of Medical Oncology, Mount Vernon Cancer Center, London HA6 2RN, UK; dr.antoniou@gmail.com

**Keywords:** NSCLC, MET, targeted therapy, pharmacogenomics, predictive biomarker

## Abstract

**Introduction**: Non-small cell lung cancer (NSCLC) is the second most common cancer globally. The mesenchymal-epithelial transition (MET) proto-oncogene can be targeted in NSCLC patients. **Methods**: We performed a literature search on PubMed in December 2019 for studies on MET inhibitors and NSCLC. Phase II and III clinical trials published in English between 2014 and 2019 were selected. **Results**: Data on MET inhibitors (tivantinib, cabozantinib, and crizotinib) and anti-MET antibodies (emibetuzumab and onartuzumab) are reported in the text. **Conclusion**: Emibetuzumab could be used for NSCLC cases with high *MET* expression. Further, studies on onartuzumab failed to prove its efficacy, while the results of tivantinib trials were clinically but not statistically significant. Additionally, cabozantinib was effective, but adverse reactions were common, and crizotinib was generally well-tolerated.

## 1. Introduction

Non-small cell lung cancer (NSCLC) is the second most common cancer in both men and women and the most common cause of death in the United States (one-quarter of all cancer deaths) [1]. The 2017 World Health Organization histological classification categorizes NSCLC into adenocarcinoma, squamous cell carcinoma, large cell carcinoma, and others [2]. There are many risk factors for NSCLC, such as substance exposure, tobacco consumption, chronic lung disease, radioactive exposure, family history of cancer, human immunodeficiency virus infection, and genetic factors [3].

The most common molecular mechanisms underlying the development of NSCLC differ among ethnic groups and with smoking status [3]. Further, 75% of lung adenocarcinomas are caused by genetic alterations promoting the RTK/RAS/RAF (receptor tyrosine kinase, rat sarcoma virus, and serine/threonine-protein kinase, respectively) signaling pathway, including Kirsten rat sarcoma (*KRAS*, 32%), epidermal growth factor receptor (*EGFR*, 11%), v-Raf murine sarcoma viral oncogene homolog B (*BRAF*, 7%), and mesenchymal-epithelial transition (*MET*) exon 14 skipping mutations (4.3%). The common alterations in squamous cell carcinoma include alterations in the RTK (26%), RAS (24%), and phosphoinositide 3-kinase (PI3K) pathways (47%); however, *MET* exon 14 skipping mutation does not qualify as one of the most common molecular mechanisms for its occurrence [4].

**Figure 1:**
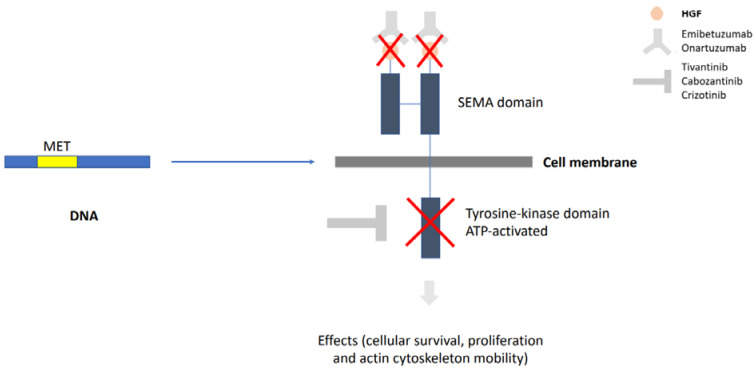
*MET* is a proto-oncogene that, when hyperactivated, interferes with cell survival and proliferation. The transmembrane protein encoded by *MET* is composed of an extracellular domain and an intracellular domain. The first is targeted by anti-MET antibodies, while the second is targeted by tyrosine kinase inhibitors. MET, mesenchymal-epithelial transition.

*MET* is a proto-oncogene that encodes a transmembrane RTK activated by an endogenous ligand, scatter factor, or hepatocyte growth factor (HGF). Its activation ultimately leads to the activation of signaling pathways, such as mitogen-activated protein kinase (MAPK) and PI3K/AKT (serine/threonine-specific protein kinase), transcription proteins, and nuclear factor κB. *MET* mutation, amplification, and/or overexpression leads to dysregulation of cell proliferation, apoptosis, and migration, which are related to NSCLC occurrence [5]. This mechanism can be seen in Figure 1.

Exon 14 skipping mutations are important molecular drivers in NSCLC and can be evaluated by new generation sequencing. As for *MET* amplifications, they are typically present in 2 to 5% of newly diagnosed adenocarcinomas, presenting higher incidence in NSCLC patients following erlotinib/gefitinib treatment (5 to 22%) and are also common in NSCLC brain metastasis. They can be evaluated by fluorescent in-situ hybridization (FISH), which uses fluorescence-marked DNA probes directly on the histological sample, analyzing multiple cells at once. *MET* rearrangements are less common than the other *MET* activation mechanisms, and it is most likely due to constitutive dimerization [5]. MET/HGF hyperactivation is also related to resistance to EGFR-TKIs (tyrosine kinase inhibitors); HGF promotes the clonal selection of c-MET amplified tumor subpopulations, which confers them substantial growth advantage and invasive potential [6]. This manuscript has focused on *MET* mutations, their inhibitors, and their use in the treatment of advanced NSCLC.

## 2. Materials and Methods

An integrative review was performed using the studies obtained from the literature search. The PubMed database was searched in May 2020. The following search terms were used in combination: “*MET* inhibitors” and “Non-Small Cell Lung Cancer”. The inclusion criteria were as follows: phase II and III clinical trials on *MET* inhibitors and anti-*MET* antibodies published in English between 2014 and 2020. Data were presented as mean values. In the Future Perspectives section, ten current studies found through the NIH’s ClinicalTrials.gov database on the topic of *MET* inhibitors and anti-*MET* antibodies are mentioned. The authors analyzed the data, statistical relevance, and risk of bias for each article in order to make a statement.

## 3. Results

Advanced NSCLC treatment had several improvements in this field over the last decade. We have discussed the main *MET* inhibitors regarding their clinical trials and tumor effects in the following paragraphs, divided by each cancer drug section (Table 2).

### 3.1. Emibetuzumab

Emibetuzumab (LY2875358) is a humanized IgG4 monoclonal bivalent MET antibody. It binds to MET ECD-Fc (Fc region of the extracellular domain) and does not trigger any functional agonist activities. The epitope of emibetuzumab is the region of the MET molecule that usually binds to hepatocyte growth factor-beta (HGFβ). Therefore, this drug prevents HGF from binding to MET. It also causes internalization and degradation of the MET receptors. These mechanisms result in the blocking of ligand-dependent and independent HGF/*MET* signaling [7].

Scagliotti et al. (2019) conducted a multicenter, randomized controlled, open-label, phase II study on erlotinib (150 mg QD) plus emibetuzumab (750 mg Q2W) as first-line treatment for *EGFR*-mutation-positive stage IV NSCLC patients. They presented data on progression-free survival (PFS), overall survival (OS), overall response rate (ORR), safety, pharmacokinetics, and exploratory analysis of *MET* expression. No statistically significant difference in the median PFS was observed in the intent-to-treat population (9.3 months for erlotinib plus emibetuzumab versus (vs.) 9.5 months for erlotinib; hazard ratio (HR) = 0.89; 95% confidence interval (CI): 0.64–1.23), but high *MET* expression (*MET* 3+ (positive) in ≥ 90% of tumor cells) was shown to be related to poor prognosis, with an improvement of 15.3 months observed in the median PFS in the erlotinib plus emibetuzumab group. The median OS (*p*-value = 0.24) showed no statistically significant trend, while the ORR for all randomized patients was 84.5% (90% CI: 75.7–91.1), including the complete responses in the combination group. Erlotinib plus emibetuzumab, although well-tolerated, led to more peripheral edema and mucositis than erlotinib by approximately 10%. Other adverse effects had similar frequencies in both study arms; therefore, emibetuzumab has a favorable safety profile. Nevertheless, doctors must consider the impact peripheral edema and mucositis, apparently mild symptoms, may have on each patient’s quality of life, in order to guarantee optimal clinical decision-making [8].

### 3.2. Onartuzumab

Onartuzumab (MetMAb) is a recombinant humanized monoclonal monovalent anti-MET antibody. This molecule consists of one single humanized antigen-binding fragment (Fab) bound to a constant domain fragment (Fc). Its Fab region binds to blades 4, 5, and 6 of the extracellular β-propeller “Sema” domain of c-MET, mainly through hydrogen interactions. The binding of onartuzumab in this site of the receptor blocks HGFα binding. The fact that onartuzumab is a monoclonal antibody prevents MET dimerization and, therefore, inhibits the activation of *MET*-related signaling pathways [9].

Han et al. (2016) analyzed the effect of onartuzumab on exposure-response and tumor growth inhibition as a second- and third-line treatment for recurrent NSCLC. This study was a phase III clinical trial that compared erlotinib (150 mg/day) vs. erlotinib plus onartuzumab. It did not meet its primary endpoint of OS and aimed to analyze whether higher doses than the phase III dose (15 mg/kg once every 3 weeks) were effective without increasing toxicity. Of note, exposure-response analysis might be confounded by disease severity and the observation of prognostic factors. The highest quartile of the erlotinib plus onartuzumab arm was associated with a longer PFS (median of 4.37 months in the highest quartile and 2.50 months in the other three quartiles) than the other quartiles and the placebo arm (2.50 months). Exploratory logistic regression showed that statistically significant infusion reactions and peripheral edema were associated with onartuzumab exposure [10].

Hirsch et al. (2016) conducted a phase II, placebo-controlled clinical trial of onartuzumab (15 mg/kg intravenous (IV) on day 1 of each 21-day cycle) plus paclitaxel (200 mg/m² IV on day 1 of each 21-day cycle) plus carboplatin or cisplatin for advanced squamous NSCLC to determine its efficacy and safety. Onartuzumab did not confer any clinical benefit in the intent-to-treat population or the *MET* immunohistochemistry-positive (IHC+) population. It is important to add that IHC is a complementary exam to pathological diagnosis, which uses the antigen-antibody binding mechanism. The primary endpoint was PFS, which was similar between the treatment arms in the intent-to-treat (HR: 0.95; 95% CI: 0.63–1.43) and *MET*-IHC+ populations (HR: 1.27; 95% CI: 0.69–2.32). The results were also similar for OS and ORR. Grade 3 to 5 adverse effects were more frequently observed in the onartuzumab arm than in the placebo arm by 5%: neutropenia (14.8% vs. 5.8%, respectively) and pulmonary embolism (5.6% vs. 0%, respectively). Four patients died in each treatment group: the causes of death were pneumonitis, pneumonia, cardiac failure, and unexplained in the combined arm and hemorrhage, cardiac arrest, hemoptysis, and febrile neutropenia in the placebo arm. In conclusion, even though onartuzumab is such a specific antibody, it still presents adverse events that should be taken into consideration in clinical decision-making [11].

Wakelee et al. (2016) used a placebo-controlled phase II trial to compare the efficacy and safety of onartuzumab (15 mg/kg IV every 3 weeks) combined with first-line bevacizumab (cohort 1) or pemetrexed-based (cohort 2) chemotherapy in stage IIIB/IV non-squamous NSCLC. No benefits were shown with the addition of onartuzumab to conventional chemotherapy, regardless of *MET* status (IHC-positive or negative). The PFS endpoint in the intent-to-treat group was numerically worse in the onartuzumab arm of the *MET*+ subgroup of cohort 1. The median PFS in cohort 1 was 5 months (95% CI: 4.7–6.9) in the onartuzumab arm and 6.8 months (95% CI: 4.9–8.8) in the placebo arm, and in the *MET*+ group, 4.8 months and 6.9 months, respectively. The median PFS in cohort 2 was 4.9 months (95% CI: 4.4–5.9) for the onartuzumab arm and 5.1 (95% CI: 4.6–7.0) months for the placebo arm, and in the *MET*+ group, 5 months for both arms. In the stratified analysis, the OS was similar in the onartuzumab and placebo arms in cohort 1 (HR: 1.34; *p*-value = 0.352) and 2 (HR: 1.15; *p*-value = 0.591). The same was observed in the stratified analysis of ORR, with no significant difference between the treatment arms. Adverse effects were frequently reported in the patients treated with onartuzumab, such as peripheral edema (30% vs. 3% in the bevacizumab cohort; 48% vs. 14% in the pemetrexed cohort) and venous thromboembolic events. At this point, it must be reminded that interactions can happen between drugs when they are combined in the treatment scheme. Both onartuzumab and bevacizumab are monoclonal antibodies; however, they have different targets (MET and vascular endothelial growth factor, respectively) and, therefore, have different anti-tumor activities and, when combined, can have a stronger anti-tumoral action; however, they can also cause more adverse events. On that note, more studies verifying the interaction between these two drugs should be conducted to analyze whether if using the combined treatment would bring clinical and statistical benefits [12].

A phase III randomized trial conducted by Spiegel et al. (2016) compared the efficacy and safety of onartuzumab (15 mg/kg IV on day 1 of each 21-day cycle) plus erlotinib (150 mg/day) vs. erlotinib (150 mg/day) alone in locally advanced previously treated stage IIIB/IV NSCLC. This study evaluated the OS, PFS, ORR, biomarkers, and safety of the treatment. The median OS was 6.8 in the onartuzumab arm vs. 9.1 months in the placebo arm (HR: 1.27; 95% CI: 0.98–1.65; *p*-value = 0.67), and the former had more deaths (52% vs. 46%) than the latter; the median PFS was 2.7 vs. 2.6 months, respectively (stratified HR: 0.99; 95% CI: 0.81–1.20; *p*-value = 0.92); the ORR was 8.4% (onartuzumab) vs. 9.6% (placebo), respectively. *MET* FISH analysis showed no benefit of onartuzumab, while *EGFR*-mutated patients showed a trend toward short OS when treated with onartuzumab (HR: 4.68; 95% CI: 0.97–22.63). In total, grade 3 to 5 adverse effects were more frequent in the onartuzumab (56%) than in the placebo arm (51.2%); some examples were dyspnea (5.2% vs. 4.5%), hypoalbuminemia (4.0% vs. 0%), venous thrombotic events (3.6% vs. 0.8%), and arterial thrombotic events (2.4% vs. 0.8%); while grade 1 or 2 events, such as rash (39%), diarrhea (39%), dermatitis acneiform (32%), decreased appetite (32%), and fatigue (30%), were similar in both groups [13]. The aforementioned efficacy results for MET antibodies can be seen in Table 1.

### 3.3. Tivantinib

Tivantinib (ARQ 197) is a small-molecule, non-adenosine triphosphate (ATP)-competitive MET inhibitor. This drug is highly selective, binding to MET only in its inactive state and causing the stabilization of the inactive molecule. The result of this is an inhibition of both intrinsic and ligand-mediated *MET* autophosphorylation, which halts the activation of *MET*-dependent signaling pathways [14,15]. In terms of pharmacokinetics, tivantinib has a half-life of 29 min and oral bioavailability of more than 20%. The metabolism of this drug involves the CYP2C19 (cytochrome P450 2C19) and CYP3A4 (cytochrome P450 3A4) pathways, and its elimination occurs through the kidneys and the digestive tract [16].

Scagliotti et al. (2015) conducted a multinational, randomized, double-blind, placebo-controlled phase III clinical trial of erlotinib (150 mg/day) plus tivantinib (360 mg twice/day) vs. erlotinib (150 mg/day) alone in previously treated patients with locally advanced or metastatic non-squamous NSCLC. The study aimed to compare the OS, PFS, and safety between the two groups. The median OS did not improve in the combined arm compared to the monotherapy arm (8.5 vs. 7.8 months, respectively; HR = 0.98; 95% CI: 0.62–0.89; *p*-value < 0.001), but exploratory analysis suggested improvement in OS in patients with high *MET* expression, defined as membranous staining intensity ≥ 2 in ≥ 50% of tumor cells upon IHC analysis (HR: 0.70; 95% CI: 0.49–1.01). Median PFS increased in the tivantinib group from 1.9 to 3.6 months (HR: 0.74; 95% CI: 0.64-0.85; *p*-value < 0.001). The most common adverse effects were rash (33.1% in the combined arm vs. 37.3% in the erlotinib arm), diarrhea (34.6% vs. 41%, respectively), asthenia (43.5% vs. 38.1%, respectively), and neutropenia (grade 3 to 4; 8.5% vs. 0.8%, respectively). Overall, it might be said that some adverse events, such as rash and diarrhea, had lower frequencies in the combined arm, making it a positive aspect in favor of tivantinib [17].

Scagliotti et al. (2017) carried out a phase III clinical trial comparing erlotinib (150 mg/day) plus tivantinib (360 mg twice/day) vs. erlotinib alone (150 mg/day) for *EGFR*-mutant NSCLC. The PFS, OS, and safety were analyzed. The median PFS improved in the combination group, from 7.5 months to 13 months (HR: 0.49; 95% CI: 0.31–0.77). The median OS was 25.5 months in the erlotinib plus tivantinib group vs. 20.3 months in the erlotinib arm (HR: 0.68; 95% CI: 0.43–1.08). The most common adverse effects were diarrhea, rash, and asthenia; neutropenia and febrile neutropenia were more common in the erlotinib plus tivantinib group than in the other groups. All in all, adverse events, such as rash and diarrhea, presented higher frequencies in the combined arm, contradicting the safety results by Scagliotti et al. (2015) [18].

Gerber et al. (2018) conducted a randomized phase II study of erlotinib (150 mg/day) plus tivantinib (360 mg twice/day) vs. single-agent chemotherapy (investigator’s choice of pemetrexed, docetaxel, or gemcitabine) in previously treated *KRAS*-mutant advanced NSCLC. The median PFS was 1.7 months for the first group vs. 4.3 months for the second, but this result was not statistically significant (HR: 1.19; 95% CI: 0.71–1.97; *p*-value = 0.50). There was no difference in the OS (HR: 1.20; 95% CI: 0.76–1.88; *p*-value = 0.44). There were no partial responses or increased frequency of dermatological toxicities in the erlotinib plus tivantinib arm, whereas there were four partial responses and increased frequency of adverse effects, such as cytopenia, nausea, fatigue, and alopecia in the chemotherapy arm. Therefore, the usage of erlotinib plus tivantinib showed statistically significant improvements neither in PFS nor in OS; however, adverse events except dermatologic toxicities occurred at a lower frequency [19].

The ATTENTION study is a randomized, double-blind, placebo-controlled, phase III trial that compares erlotinib with or without tivantinib in Asian patients with previously treated IIIB/IV non-squamous NSCLC harboring wild-type EGFR. Enrollment had to stop earlier than imagined, with 307 rather than 460 patients, because there was a higher rate of interstitial lung disease in the tivantinib (14 patients, 3 deaths) than the placebo group (6 patients, 0 deaths). Results from enrolled patients showed a statistically insignificant difference in median OS (12.7 months for tivantinib vs. 11.1 months for placebo, HR = 0.891, *p* = 0.427). Median PFS was higher in the tivantinib group (2.9 months vs. 2.0 months for placebo, HR = 0.719, *p* = 0.019), which also presented more grade 3 or higher hematological adverse events, such as neutropenia (24.3% vs. 0.7% for placebo), leukopenia (18.4% vs. 0.7%), febrile neutropenia (13.8% vs. 0%), and anemia (13.2% vs. 6.6%). However, the ATTENTION study lacks statistical validation because of the early need for stopping enrollment [20].

### 3.4. Cabozantinib

Cabozantinib (XL184) is an ATP-competitive inhibitor of a wide range of kinase receptors (such as MET, vascular endothelial growth factor receptor [VEGFR], protein encoded by the rearranged during transfection oncogene [RET], tyrosine-protein kinase receptor UFO [AXL], amongst many others), blocking their autophosphorylation, which stops them from activating intracellular signaling pathways. This drug shows a high potency of inhibition for MET through a reversible effect. In terms of pharmacokinetics, this drug has a maximum-tolerated dose of 175 mg per day, a median half-life of 91 h, and a 20% rate of penetration of the blood-brain barrier [21]. 

Drilon et al. (2016) conducted a phase II, single-arm trial of cabozantinib (60 mg/day) in patients with advanced, metastatic, or unresectable *RET*-rearranged lung cancer. The ORR, PFS, OS, and toxicity were analyzed. Seven out of 25 response-evaluable patients had partial responses (ORR = 28%, 95% CI: 12–49%). The median PFS and median OS of the cabozantinib group were lower than that of the single-agent TKI therapy group. The most common adverse effects were asymptomatic lipase elevation (15%), increased alanine aminotransferase (8%), increased aspartate aminotransferase (8%), thrombocytopenia (8%), and hypophosphatemia (8%) [22].

Neal et al. (2017) carried out a three-arm, randomized phase II clinical trial to determine the efficacy of erlotinib (150 mg/day), cabozantinib (60 mg/day), and erlotinib (150 mg/day) plus cabozantinib (40 mg/day) as second- or third-line treatment for patients with non-squamous *EGFR* wild-type NSCLC. The study analyzed PFS, HR, OS, radiographic response, and adverse events. The PFS had significantly improved in the cabozantinib arm (4.3 months; HR: 0.39; *p*-value < 0.05; 80% CI: 0.27–0.55) and in the combined arm (4.7 months; HR: 0.37; *p*-value < 0.05; 80% CI: 0.25–0.53). The most common grade 3 or 4 adverse events were diarrhea (8% in the erlotinib group, 8% in the cabozantinib group, and 28% in the combined group), hypertension (none vs. 25% vs. 3%, respectively), fatigue (13% vs. 15% vs. 15%, respectively), oral mucositis (none vs. 10% vs. 3%, respectively), and thromboembolic events (none vs. 8% vs. 5%, respectively). One death due to respiratory failure occurred in the cabozantinib group, and one death due to pneumonitis occurred in the combined arm. There was no correlation between *MET* IHC status and PFS and treatment with cabozantinib. In conclusion, this study showed that while cabozantinib produced a promising increase in PFS, it also increased the occurrence of grade 3 and 4 adverse events [23].

Schöffski et al. (2017) conducted a multicenter phase II randomized discontinuation trial of cabozantinib (100 mg/day) in patients with advanced, recurrent, or metastatic solid tumors, including NSCLC (N = 60). The endpoints were ORR at week 12 and PFS. The disease control rate (DCR) was 38.3% (95% CI: 26.1–51.8) for NSCLC, ORR was 10% (95% CI: 4.4–20.3), and median PFS was 2.4 months (95% CI: 1.4–2.66; HR: 0.94; *p*-value = 0.93), but the PFS was not statistically significant. It is also important to highlight that this study did not evaluate the patient’s *MET* expression or made any distinctions if different results were achieved for patients with higher or lower *MET* expression [24].

In a 12-week study conducted by Hellerstedt et al. (2018), as part of phase II randomized, double-blinded discontinuation trial with cohorts of nine tumor types, the effects of cabozantinib 100 mg/day vs. placebo on patients with stable lung cancer who had received a median of two prior lines of therapy were analyzed, in terms of PFS and objective response rate. Due to unavailable regulatory consent in some of the recruitment sites, the researchers did not perform the detection of MET, AXL, and RET. The cabozantinib group had an overall DCR of 38%, ORR at week 12 was 10% (six patients confirmed partial response), and 64% of these patients showed tumor regression, including those with *KRAS* and *EGFR* mutations. There was no statistically significant difference in the median PFS values between the two groups (from 4.2 months at week 0 to 2.4 months at week 12 in the cabozantinib and placebo groups). The most common adverse effects were fatigue (13%), palmar-plantar erythrodysesthesia (10%), diarrhea (7%), hypertension (7%), and asthenia (5%) [25].

### 3.5. Crizotinib

Crizotinib (PF-02341066) is a selective inhibitor of receptors, such as anaplastic lymphoma kinase (ALK), MET, and proto-oncogene tyrosine-protein kinase ROS (ROS1). This drug is considered a class I kinase inhibitor because it binds to the ATP-binding site of the receptors by forming a U-shaped loop that stabilizes the catalytically inactive conformation of each receptor. A key part of the binding of crizotinib to MET is the establishment of a π−π interaction, with the Tyr-1230 residue of the protein, in a dose-dependent manner [26]. In terms of pharmacokinetics, the peak plasma concentration of crizotinib is around 109 ng/mL, achieved from 2 to 6 h after oral administration of a single 250 mg dose, with a half-life of 29 to 51 h. One of the main metabolites of crizotinib, named crizotinib lactam, has a shorter half-life (20 h) and inhibitory activity on MET receptors, although this inhibition is four times less potent than the original drug [27].

Wu et al. (2018) conducted a phase II study of crizotinib (250 mg twice/day) in East Asian patients with ROS1-positive advanced NSCLC and analyzed the ORR, PFS, and safety of crizotinib. The ORR was 71.7% (95% CI: 63–79.3), with 17 complete to 74 partial responses. The median duration of response (DOR) was 19.7 months (95% CI: 14.1–not reached). Median PFS was 15.9 months (95% CI: 12.9–24.0), and the drug’s safety profile remained consistent with previous reports, with no addition of new safety signals; crizotinib was generally well-tolerated [28].

Sargis and Salgia (2015) evaluated seven consecutive patients with NSCLC who were being treated with crizotinib. Four out of five males presented with primary hypogonadism, four out of five with mildly elevated prolactin, three out of seven with hypocalcemia, and five out of seven with elevated levels of insulin growth factor 1 (IGF-1). Therefore, the cellular interaction between *MET* and IGF-1 signaling led to endocrine disruptions in male patients treated with crizotinib [29].

Hida et al. (2017) compared alectinib (300 mg twice/day) and crizotinib (250 mg twice/day) in patients with ALK-positive NSCLC in a randomized, open-label, randomized phase III clinical trial. Alectinib is a potent and highly selective central nervous system (CNS)-active ALK inhibitor. The parameters evaluated were PFS and safety. The median PFS was not reached in the second interim analysis for alectinib, and it was reached for crizotinib (10.2 months). Grade 3 or 4 adverse effects were more frequent in the crizotinib arm (54%) than in the alectinib arm (27%). More patients in the crizotinib group than in the alectinib arm interrupted or discontinued the treatment due to adverse events [30].

Gadgeel et al. (2018) compared alectinib (600 mg twice/day) and crizotinib (250 mg twice/day) in a randomized phase III ALEX study, with a special focus on central nervous system (CNS) metastases. The study population included patients with treatment-naive ALK+ NSCLC. This study aimed to compare the PFS, ORR, and time to CNS metastasis between the two drugs. The investigator-assessed PFS of the patients in the alectinib group was similar between patients with or without baseline CNS metastases, regardless of prior radiotherapy. Time to CNS progression was longer in the alectinib arm than in the crizotinib arm and could be compared to that in patients without baseline CNS metastases. CNS ORR was higher in the alectinib group (85.7%) than in the crizotinib group (78.6%) and higher among patients who underwent prior radiotherapy than among those who did not. Additionally, the study showed that the group of patients who received crizotinib presented more CNS metastasis. In conclusion, alectinib could be considered more effective than crizotinib in combating CNS metastases and delaying its occurrence; however, it is important to add that this study was not specific to patients with MET alterations, although it adds important information regarding crizotinib [31].

### 3.6. Capmatinib

Capmatinib (INCB28060) is a small-molecule, ATP-competitive, and highly selective inhibitor of the c-MET receptor. This drug binds to MET via an aromatic stacking interaction with the Y1230 residue of the protein, and this, like crizotinib, stabilizes the kinase loop of MET in its inactive state [32]. Capmatinib is more potent than crizotinib and is especially potent in c-MET molecules that have specific mutations, such as exon 14 deletions [33]. In terms of pharmacokinetics, a study found that a 200 mg dose of capmatinib had a mean half-life of 4.82 h and a mean peak plasma concentration of 1.02 ng/mL, which was achieved in a median of 2 h after administration [34]. 

The GEOMETRY mono-1 phase II clinical trial tested the efficacy of daily administration of 400 mg of capmatinib in patients with various MET alterations, and the principal subgroups were composed either of patients with MET exon 14-mutated NSCLC who received 2 prior lines of treatment (cohort 4) or treatment-naïve patients (cohort 5b). The best results were achieved in cohort 5b, regardless of gene copy number (GCN); ORR was 71.4%, and the median duration of response was 9.13 months. In cohort 4, regardless of GCN, PFS was 5.42 months, and the ORR was 39.1%. However, the researchers of this trial reinforced that data on durability, although promising, was still immature at the time of the analysis. Most adverse events were grade 1 or 2. Across all cohorts, the principal ones were peripheral edema (49.2%), nausea (43.2%), and vomiting (28.3%), which could compromise the patient’s quality of life [33].

Wu et al. conducted a phase II clinical trial, investigating the effects of capmatinib (400 mg twice a day) associated with gefitinib (250 mg once a day) in patients with activating MET mutations who had a background of tumor progression despite being administered anti-EGFR treatment. The overall DCR in these patients was 73%, and the median DOR was 5.6 months (95% CI 3.8 to 7.2 months). In terms of ORR, the most promising results were achieved in the subgroup of patients who had MET gene amplification with GCN ≥ 6 (ORR = 47%), while the entire group of patients, regardless of MET status, achieved an ORR of 29%. However, PFS was similar in the group with all the patients (5.5 months, 95% CI 3.8 to 5.6 months) and the GCN ≥ 6 (5.49 months, 95% CI 4.21 to 7.29 months). In terms of safety, 87% of all patients reported treatment-related adverse events, such as nausea (28%), peripheral edema (22%), and rash (20%) [3]. It is also important to consider the possibilities of drug-drug interactions; however, there were no significant ones reported between capmatinib and EGFR-TKIs, such as gefitinib, which was approved by the FDA as first-line treatment of metastatic NSCLC in 2015 [35]. The aforementioned efficacy results for MET inhibitors can be seen in Table 2. 

## 4. Discussion

Despite lung cancer carries an ominous prognosis, currently available treatments for advanced NSCLC include traditional chemotherapy, radiotherapy, targeted therapies, and immunotherapy. Oncogene addiction drives the efficacy of targeted therapy. Tumor molecular heterogeneity, however, poses a priori a challenge for the future to identify selective population beneficiaries.

Studies have shown that MET expression is associated with poor prognosis in lung cancer. In line with the aforementioned studies, emibetuzumab offers a reasonable option for highly MET expressing tumors, once careful consideration has been given on the cost-benefit relationship due to the increased toxicity [7,8]. However, clinicians should never ignore what repercussions novel therapies can have on the patients’ quality of life. Emibetuzumab shows promising results that may be beneficial to some patients at first, but adverse events could become worse over time and eventually contraindicate the use of this drug to classic treatment regimens.

Onartuzumab is another treatment option. We found that it has not shown very optimistic results; only a few patients showed improvements in their PFS, and adverse events occurred frequently with this drug. Therefore, these studies fail to prove the drug’s absolute efficacy in combination with erlotinib or platinum-based chemotherapy. When used in association with bevacizumab, the results were even worse. In this context, many other significant studies are needed to assess the efficacy and safety of this medicine [10,11,12,13]. In conclusion, onartuzumab lacks more studies showing an improvement in PFS, while it remains with significant evidence of harmful adverse events and could worsen the quality of life.

Studies on tivantinib showed an improvement in PFS and OS, but adverse reactions were common. The benefit that appeared in these studies was clinically significant but did not reach statistical significance in all cases. Given the gravity of the adverse events and their high incidence, the benefit of this treatment in comparison to that of the regular protocol should be considered, always aiming at improving quality of life [17,18,19].

Cabozantinib seemed to be less effective than TKIs in Drilon et al. (2016). On the other hand, Neal et al. (2017) presented a different result, with higher PFS achieved with cabozatinib, whether alone or combined with erlotinib, compared to erlotinib alone. Therefore, this multikinase inhibitor could be useful, especially considering that MET hyperexpression is a common resistance mechanism. The number of adverse events was significant. In general, cabozantinib improved OS and PFS, but not all results were statistically significant. In this context, cabozantinib seems to be a suitable treatment option, but, once again, more studies are needed to evaluate with more certainty which treatment is the best option to extend patients’ lifetime and improve the quality of life, considering all benefits and limitations of each treatment [22,23,24,25].

Crizotinib is generally well-tolerated, although grade 3 or 4 adverse events occurred in some cases, leading some patients to interrupt treatment, and presented good results as it improved PFS and OS in most studies. However, it is important to follow patients treated with crizotinib for possible endocrine disorders, especially male patients. Crizotinib treatment could be beneficial when considering CNS metastases as well. In conclusion, clinicians should evaluate the clinical condition of each patient to decide if the usage of crizotinib would be beneficial or harmful [28,29,30,31].

Among the assessed drugs, capmatinib is the one that has been most recently approved by the FDA, and it still lacks studies to analyze even more this drug’s efficacy and safety. The two analyzed trials showed promising results, especially in treatment-naïve patients with MET exon 14-mutated NSCLC. Nevertheless, the data on durability was still immature when analyzed. The second study also presented a positive perspective for the use of capmatinib to treat NSCLC, with more significant results in patients with a higher number of gene copy number. Adverse events were frequent in both studies, so the scientific community must keep researching to establish if the drug’s highlights overcome eventual harm that could be made and to which patients it would be more strongly indicated [33,34,35].

When it comes to immunotherapy, patients with mutated MET have a low ORR to these drugs (17%). However, MET expression is relevant to immunotherapy because it was found to be a predictor of response to different types of immunotherapy agents. High expression of MET was correlated with poorer outcomes in anti-EGFR treatment (*p* = 0.053). On the contrary, in programmed death ligand 1 (PD-L1) targeted therapy, a study found that OS was about 25% superior in patients with overexpressed MET. This opens up a promising perspective because studies show that around 60% of patients with MET exon 14 skipping alterations are positive for PD-L1 [36].

It is important to highlight that each patient is different. Clinical history, molecular profiling of the tumor, and genetic characteristics of the patient should always be taken into consideration when choosing the right treatment. Not every patient will respond in the same way to a specific medication, and it is essential to evaluate the clinical development of each patient and adjust the dosage and duration of medication over time, given possible alterations in response, considering the benefits and adverse effects and advances in scientific knowledge.

## 5. Conclusions

### Conclusions and Future Perspectives

*MET* inhibitors are a group of drugs that could help NSCLC patients with MET alterations, especially exon 14 skipping mutation, present in 2–3% of lung cancers [37]. Overexpression, amplification, and point mutations of *MET* seem to be more challenging subgroups to target. These target medications could be associated with *EGFR* inhibitors, for example, to treat certain patients (e.g., EGFR-TKI resistance) [38]. There are ongoing clinical trials of cabozantinib and crizotinib, which are available in the US clinical trials register. Of note, other drugs are being studied as well, such as capmatinib, a highly potent and selective TKI [39], tepotinib, a selective MET-TKI [40], osimertinib, a third-generation EGFR-TKI designed to overcome *T790M*-mediated resistance [41], savolitinib, a novel small-molecule selective cMET inhibitor [42], and TPX-0022, a novel MET/CSF1R/SRC (MET/Colony stimulating factor 1 receptor/Proto-oncogene tyrosine-protein kinase Src) inhibitor. A large number of clinical trials are being conducted worldwide. Some relevant studies recovered from these databases are presented in Table 3 [43,44]. Given the importance of this subject and its capacity to improve the way health professionals deal with NSCLC, the authors believe that in 5–10 years, this field will evolve with more research about the efficacy and safety of these drugs. These drugs could be alternatives for NSCLC treatment of patients with *MET* mutations and/or EGFR-TKI resistance, resulting in improvements in the way patients with NSCLC are treated, their survival, and their quality of life during and after treatment.

## Figures and Tables

**Table 1:** Met antibodies clinical trials in combination with chemotherapy and targeted therapies.

Drugs	PFS (Median, Months)	OS (Median, Months)	DOR (Median, Months)	ORR (Median, %)	DCR (Median, %)	References
**Emibetuzumab + erlotinib**	9.30 *	34.40 *	9.50	84.50	10,000%	[8]
**Erlotinib**	9.50 *	25.40 *	11.10	65.70	98.60	[8]
**Onartuzumab + erlotinib (highest quartile)**	4.37	**	**	**	**	[10]
**Placebo + erlotinib**	2.59	**	**	**	**	[10]
**Onartuzumab + paclitaxel + carboplatin/cisplatin**	4.90	9.10	**	40.00 *	**	[11]
**Placebo + paclitaxel + carboplatin/cisplatin**	4.90	8.50	**	43.40 *	**	[11]
**Onartuzumab + bevacizumab-based chemotherapy**	5.00	***	**	51.50 *	**	[12]
**Placebo + bevacizumab-based chemotherapy**	6.80	16.50	**	44.80 *	**	[12]
**Onartuzumab + pemetrexed-based chemotherapy**	4.90	8.50	**	28.60 *	**	[12]
**Placebo + pemetrexed-based chemotherapy**	5.10	13.70	**	36.10 *	**	[12]
**Onartuzumab + erlotinib**	2.70 *	6.80	**	8.40	**	[13]
**Placebo + erlotinib**	2.60 *	9.10	**	9.60	**	[13]

PFS = progression-free survival; OS = overall survival; ORR = overall response rate; DCR = disease control rate; DOR = duration of response; * = the difference between the groups is not statistically significant; ** = unavailable data; *** = this value has not yet been reached. The data are based on the results of the intent-to-treat population in studies approaching anti-MET (mesenchymal-epithelial transition) antibodies.

**Table 2:** Met inhibitors clinical trials in combination with targeted therapies.

Drugs	PFS (Median, Months)	OS (Median, Months)	DOR (Median, Months)	ORR (Median, %)	DCR (Median, %)	References
**Tivantinib + erlotinib**	3.60	8.50 *	10.10	10.30	45.80	[17]
**Placebo + erlotinib**	1.90	7.80 *	11.90	6.50	32.00	[17]
**Tivantinib + erlotinib**	13.00	25.50	12.70	60.70	**	[18]
**Placebo + erlotinib**	7.50	20.30	9.80	43.30	**	[18]
**Tivantinib + erlotinib**	1.70 *	6.80	**	0	49.00	[19]
**Single-agent chemotherapy**	4.30 *	8.50	**	4.40	62.20	[19]
**Tivantinib + erlotinib**	2.90	12.70	**	**	**	[20]
**Placebo + erlotinib**	2.00	11.10	**	**	**	[20]
**Cabozantinib**	5.50	9.90	7.00	28.00	**	[22]
**Cabozantinib**	4.30	9.20	**	**	**	[23]
**Erlotinib**	1.80	5.10	**	**	**	[23]
**Cabozantinib + erlotinib**	4.70	13.30	**	**	**	[23]
**Cabozantinib**	2.40 *	**	6.90	10.00	38.30	[24]
**Cabozantinib**	2.40 *	7.70	**	10.00	38.00	[25]
**Crizotinib**	15.90	32.50	19.70	71.70	80.30	[28]
**Crizotinib**	10.20	20.00	11.10	**	**	[30]
**Alectinib**	***	30.00	**	**	**	[30]
**Crizotinib**	10.90	**	**	**	**	[31]
**Alectinib**	34.80	**	**	**	**	[31]
**Capmatinib (cohort 4)**	5.42	**	9.72	39.10	**	[33]
**Capmatinib (cohort 5b)**	9.13	**	8.41	71.40	**	[33]
**Capmatinib + gefitinib (GCN ≥ 6)**	5.50	**	**	47.00	**	[35]
**Capmatinib + gefitinib (all patients)**	5.49	**	5.60	29.00	73.00	[35]

PFS = progression-free survival; OS = overall survival; ORR = overall response rate; DCR = disease control rate; DOR = duration of response; * = the difference between the groups is not statistically significant; ** = unavailable data; *** = this value has not yet been reached. The data are based on the results of the intent-to-treat population in studies approaching MET inhibitors.

**Table 3:** The current ongoing clinical trials.

Study Title	Status	Condition	Intervention	Phase	Locations	Enrollment	Start	End
Phase 1 Study of TPX-0022, a MET/CSF1R/SRC Inhibitor, in Patients With Advanced Solid Tumors Harboring Genetic Alterations in MET	R	Advanced solid tumors, metastatic solid tumors, *MET* gene alterations	TPX-0022	1	UC Irvine Chao Family Comprehensive Center (CA, US) UC San Diego Moores Cancer Center (CA, US) Sarah Connor Research Institute at HealthONE (CO, US) and 5 more	120	Aug 2019	Nov 2023
Study of Crizotinib for ROS1 and MET Activated Lung Cancer	NYR	Non-squamous NSCLC, stage IV NSCLC, ROS1 gene rearrangement, *MET* activating mutation, *MET* amplification	Crizotinib	2	Princess Margaret Cancer Centre (Toronto, ON, Canada)	50	Dec 2019	Jun 2025
Osimertinib Plus Savolitinib in EGFRm+/MET+ NSCLC Following Prior Osimertinib (SAVANNAH)	R	Carcinoma	Osimertinib Savolitinib	2	La Jolla, CA, US Los Angeles, CA, US) Sacramento, CA, US and 97 more	192	Jan 2019	Jul 2022
A Study of Capmatinib (INC280) in NSCLC Patients With MET Exon 14 Alterations Who Have Received Prior MET Inhibitor	R	Malignant NSCLC stage IV	Capmatinib	2	Massachusetts General Hospital (Boston, MA, US)	20	May 2016	Dec 2020
Clinical Study of Oral cMET Inhibitor INC280 in Adult Patients With EGFR Wild-type Advanced Non-small Cell Lung Cancer	R	NSCLC carcinoma	Capmatinib	2	UAMS (Little Rock, AR, US) Pacific Shores Medical Group (Long Beach, CA, US) Los Angeles Hematology/Oncology Medical Group (CA, US) and 153 more	373	Jun 2015	Sep 2022
Capmatinib in Patients With Non-small Cell Lung Cancer Harboring cMET exon14 Skipping Mutation	R	Metastatic NSCLC *MET* gene mutation	Capmatinib		Asan Medical Center (Seoul, Republic of Korea)	27	Oct 2018	Jun 2022
Tepotinib Phase II in Non-small Cell Lung Cancer (NSCLC) Harboring MET Alterations (VISION)	R	Stage IIIB/IV NSCLC with METex14 skipping alterations or *MET* amplification	Tepotinib	2	City of Hope Cancer Center (Duarte, CA, US) Torrance Health Association (Redondo Beach, CA, US) and 95 more	280	Sep 2016	Feb 2023
A Study of Tepotinib Plus Osimertinib in Epidermal Growth Factor Receptor (EGFR) Tyrosine Kinase Inhibitor (TKI) Relapsed Mesenchymal-epithelial Transition Factor (MET) Amplified Non-small Cell Lung Cancer (NSCLC)	R	NSCLC	Tepotinib Osimertinib	2	Compassionate Care Research Group Inc (Fountain Valley, CA, US) St. Louis Cancer Care (Bridgeton, MO, US) Tennessee Oncology (Nashville, TN, US)	90	Sep 2019	Mar 2022
CABozantinib in Non-Small Cell Lung Cancer (NSCLC) Patients With MET Deregulation (CABinMET)	R	NSCLC	Cabozantinib	2	AOS Giuseppe Moscati (Avellino, AV, Italy) IRCCS Oncologico Giovanni Paolo II (Bari, BA, Italy) AOU Careggi (Firenze, FI, Italy) and 17 more	25	Sep 2018	Sep 2020
Evaluating Crizotinib in the Neoadjuvant Setting in Patients With Non-small Cell Lung Cancer	R	NSCLC	Crizotinib	2	University of Colorado Denver (Aurora, CO, US)	18	Dec 2017	Oct 2021

Some of the current clinical trials on MET inhibitors [41]. R = recruiting; NYR = not yet recruiting. Enrollment is presented as an estimated number of participants. End accounts for the estimated study conclusion date.

## Abbreviations

NSCLC	Non-small cell lung cancer
RTK	Receptor tyrosine kinase
RAS	Rat sarcoma virus
RAF	Serine/threonine-protein kinase
KRAS	Kirsten rat sarcoma
EGFR	Epidermal growth factor receptor
BRAF	v-Raf murine sarcoma viral oncogene homolog B
MET	Mesenchymal-epithelial transition
PI3K	Phosphoinositide 3-kinase
HGF	Hepatocyte growth factor
MAPK	Mitogen-activated protein kinase
FISH	Fluorescent in-situ hybridization
TKI	Tyrosine-kinase inhibitors
PFS	Progression-free survival
OS	Overall survival
ORR	Overall response rate
HR	Hazard ratio
CI	Confidence interval
IV	Intravenous
IHC	Immunohistochemistry
DCR	Disease control rate
DOR	Duration of response
CNS	Central nervous system
